# Tumor necrosis factor alpha drugs in rheumatoid arthritis: systematic review and metaanalysis of efficacy and safety

**DOI:** 10.1186/1471-2474-9-52

**Published:** 2008-04-17

**Authors:** Alberto Alonso-Ruiz, Jose Ignacio Pijoan, Eukene Ansuategui, Arantxa Urkaregi, Marcelo Calabozo, Antonio Quintana

**Affiliations:** 1 Rheumatology service (Cruces Hospital), Barakaldo, Spain; 2 Clinical epidemiology section (Cruces Hospital), Baracaldo, Spain; 3 Health library (Donostia Hospital), San Sebastián, Spain; 4 Department of applied mathematics, statistics and operational research, faculty of science and technology (University of the Basque Country), Leioa, Spain; 5 Department of pharmacology, faculty of medicine and odontology (University of the Basque Country), Leioa, Spain

## Abstract

**Background:**

To analyse available evidence on the efficacy and safety of anti-TNFα drugs (infliximab, etanercept and adalimumab) for treating rheumatoid arthritis (RA).

**Methods:**

We searched systematically for randomised controlled clinical trials on treatment of RA with anti-TNFα drugs, followed by a systematic review with metaanalysis. Trials were searched from MEDLINE, EMBASE and Cochrane Library databases. The American College of Rheumatology (ACR) efficacy response criteria were used. Safety parameters provided by the trials were also assessed. Positive and undesired effects were estimated using combined relative risks (RR), number needed to treat (NNT) and number needed to harm (NNH). Heterogeneity was evaluated by Cochrane's Q and I^2 ^statistics.

**Results:**

Thirteen trials (7087 patients) met the inclusion criteria. The combined RR to achieve a therapeutic response to treatment with recommended doses of any anti-TNFα drug was 1.81 (95% CI 1.43–2.29) with a NNT of 5 (5–6) for ACR20. NNT for ACR50 [5 (5–6)] and ACR70 [7 (7–9)] were similar. Overall therapeutic effects were also similar regardless of the specific anti-TNFα drug used and when higher than recommended doses were administered. However, lower than recommended doses elicited low ACR70 responses (NNT 15). Comparison of anti-TNFα drugs plus methotrexate (MTX) with MTX alone in patients with insufficient prior responses to MTX showed NNT values of 3 for ACR20, 4 for ACR50 and 8 for ACR70. Comparison of anti-TNFα drugs with placebo showed a similar pattern. Comparisons of anti-TNFα drugs plus MTX with MTX alone in patients with no previous resistance to MTX showed somewhat lower effects. Etanercept and adalimumab administered as monotherapy showed effects similar to those of MTX. Side effects were more common among patients receiving anti-TNFα drugs than controls (overall combined NNH 27). Patients receiving infliximab were more likely to drop out because of side effects (NNH 24) and to suffer severe side effects (NNH 31), infections (NNH 10) and infusion reactions (NNH 9). Patients receiving adalimumab were also more likely to drop out because of side effects (NNH 47) and to suffer injection site reactions (NNH 22). Patients receiving etanercept were less likely to drop out because of side effects (NNH for control versus etanercept 26) but more likely to experience injection site reactions (NNH 5).

**Conclusion:**

Anti-TNFα drugs are effective in RA patients, with apparently similar results irrespective of the drug administered. Doses other than those recommended are also beneficial. The main factor influencing therapeutic efficacy is the prior response to DMARD treatment. The effect of treatment with etanercept or adalimumab does not differ from that obtained with MTX. The published safety profile for etanercept is superior but the fact that no patients are treated with higher than recommended doses requires explanation.

## Background

Rheumatoid arthritis (RA) is a chronic, systemic, inflammatory disease of the joints, which often causes joint destruction, deformity and functional impairment [1]. Early administration of disease-modifying antirheumatic drugs (DMARDs) is crucial and the use of nonsteroidal anti-inflammatory drugs and glucocorticoids remains a fundamental aspect of medical management of RA. The discovery that the macrophage-derived proinflammatory cytokine tumour necrosis factor alpha (TNFα) plays a central role in the pathogenesis of RA [2] led to the introduction of anti-TNFα drugs, a new biological DMARD class. Evidence showing that anti-TNFα drugs are very effective in RA has led to a substantial change in the treatment of this disease [3]. Three such drugs have been commercialized since 1999: infliximab, etanercept and adalimumab. Despite this relative short history, a considerable amount of information has already been accumulated [4-6]. However, many questions about this new class of drugs still remain unanswered: are all available anti-TNFα drugs equally effective; does their efficacy depend upon their being administered together with methotrexate (MTX); does efficacy depend on dose; are they more effective than MTX; are all anti-TNFα drugs equally safe; what is the efficacy/safety profile? To date, no direct "head-to-head" comparative studies of the different anti-TNFα drugs have been published. An alternative approach to answering the foregoing questions is to perform a systematic review with metaanalysis of relevant research. A metaanalysis with emphasis on the risk of cancer and infections has been reported [7]. Also, a study using an indirect comparative approach to the relative efficacies of the three anti-TNFα drugs in the treatment of RA showed no differences among them [8].

In this paper, we conduct a systematic review of randomised controlled clinical trials of anti-TNFα drugs in RA followed by a metaanalysis of the efficacy and safety of different doses of infliximab, etanercept and adalimumab.

## Methods

### Study selection criteria

We carried out a search of all randomised controlled clinical trials of anti-TNFα drugs (infliximab, etanercept or adalimumab) for treating patients with RA. Patients had to satisfy the American College of Rheumatology (ACR) criteria [9] for diagnosis and to have active disease. Trial duration had to be at least 6 months with efficacy measured by ACR response [10]. Clinical trials were excluded if they either used administration routes other than recommended or included no treatment arm with recommended doses. Only information published in the trial reports was assessed.

### Efficacy parameters

We used the ACR responses ACR20, ACR50 and ACR70 (improvements of at least 20, 50 and 70%, respectively, on a series of predetermined measures) as efficacy parameters [10].

### Safety parameters

The following safety parameters reported in the selected trials were analyzed: number of patients suffering any adverse event, withdrawals due to adverse events, serious adverse events, infections, serious infections, infusion reactions, injection-site reactions, malignancies and overall mortality.

### Search strategy

Trials were searched in scientific journals and congress conference proceedings. Information from the MEDLINE, EMBASE and Cochrane Library databases up to October 2006 was checked using a high-sensitivity strategy. The descriptors used were rheumatoid arthritis, infliximab, etanercept, adalimumab, randomised controlled trial and meta-analysis. The computerised search was completed with a manual search of reference lists from the articles retrieved and from rheumatological journal articles published in 2006 (technical details are available from the authors). There was no language restriction.

### Data extraction

Two investigators (AA-R and MC) independently examined each eligible study and extracted data. Trials with information only in abstract format were excluded. Data were extracted using an ad hoc form with key items for each trial: study design, patients' characteristics (sex, age and duration of disease evolution), patient inclusion criteria, drugs and doses used, treatment duration and ACR response and safety parameters. Special attention was paid to both inclusion criteria and clinical features of patients included in each trial, as they were deemed central aspects for assessing heterogeneity. The quality of each individual study was assessed and scored using the Jadad scale [11].

### Statistical analysis

For each single trial the relative risk (RR) of attaining an ACR response was obtained as a measure of the effect. Overall efficacy estimates (combined relative risk) for each anti-TNFα drug (as monotherapy or in association with MTX or another DMARD) compared to a control (placebo, MTX or another DMARD) were attained using the ACR20, ACR50, and ACR70 criteria as the main outcome variables. We used DerSimonian-Laird's method to estimate a random-effects model. Heterogeneity was evaluated using Cochrane's Q and I^2 ^statistics and explored via subgroup analysis. The I^2 ^statistic is calculated from Q and can be interpreted as the percentage variability in study results attributable to between-study differences [12]. The number of patients needed for experimental treatment versus control (NNT) to obtain an additional positive ACR response was also estimated [13].

We also used the RR to estimate the risk of adverse effects; and we estimated the number needed to harm (NNH), defined as the number of patients receiving active treatment that would harm one patient compared to controls [13-15].

Publication bias was assessed graphically using a funnel plot [16] and statistically evaluated by the regression symmetry test described by Egger et al. [17] and the adjusted rank correlation test proposed by Begg and Mazumdar [18]. We used the specific software Comprehensive Metaanalysis Version 2.0 for analysis and presentation of main results.

## Results

### Search results

**Figure 1:**
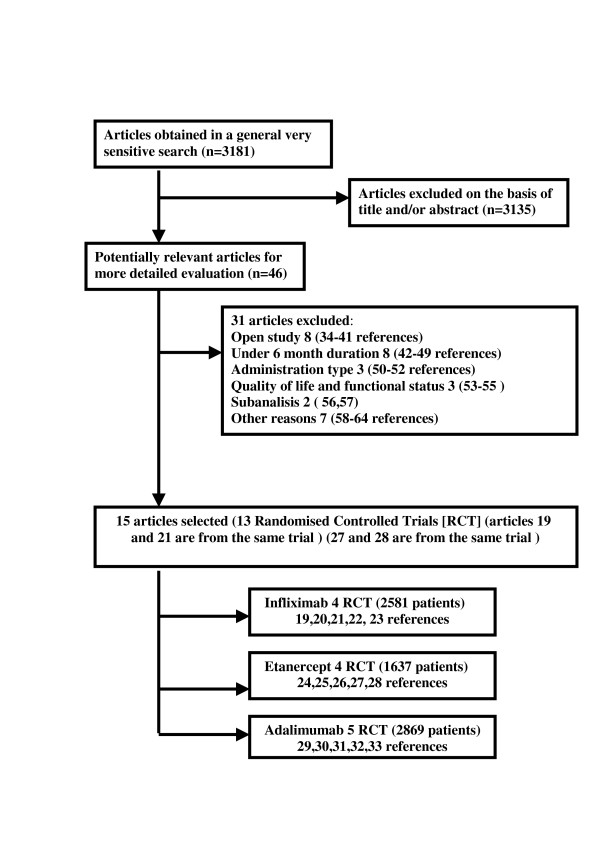
Flow chart of the selection process for inclusion of Randomised Controlled Trials (RCT) in the overview.

Of the 46 publications located [19-65], only 15 met the selection criteria and were consequently included in the metaanalysis [19-33]. The remaining 31 were excluded for several reasons [34-64] (Figure 1). The Maini trial [21] was included in the Lipsky et al. trial [19] and the van der Heijde et al. [28] and Klareskog et al. [27] trials were the same (TEMPO trial). We analyzed the entire set of 7087 patients recruited for the 13 trials selected: four using infliximab [19,20,22,23] (2581 patients), four etanercept [24-26,28] (1637 patients) and five adalimumab [29-33] (2869 patients). The methodological quality of the studies was moderate to high (3–5) except Bathon's trial [24], which had a lower Jadad score of 2 because it neither mentioned nor explained whether treatment allocation was based on a random procedure (Table 1).

**Table 1:** Summary of trials included in the metaanalysis

**Trial (reference) Comparisons Jadad's scale (J)**	**Groups and N of patients**		**Mean age (years)**	**Mean disease duration (years)**	**N of swollen joints**	**N of tender joints**	**CRP (mg/dl)**	**HAQ**	**Mean N of previous DMARDs**	**Previous response to MTX**
Lipsky et al. (19) Infliximab+MTX vs. MTX J [5]	3 mg/Kg 8 wk *	86	54	10	22	32	3.9	1.8	3.8	Insufficient
	3 mg/Kg 4 wk	86	52	9	21	31	3.5	1.7	2.6	
	10 mg/Kg 8 wk	87	54	11	23	32	3.3	1.7	2.5	
	10 mg/Kg 4 wk	81	52	12	24	34	4.2	1.7	2.5	
	MTX	88	51	11	21	31	4.0	1.7	2.5	
	total	428								
St. Clair et al. (20) Infliximab+MTX vs. MTX J [3]	3 mg/Kg 8 wk *	373	51	0,8	21	32	2.9	1,5	71%	Not previously MTX
	6 mg/Kg 8 wk	378	50	0,9	22	33	3.0	1,5	68%	
	MTX	298	50	0,9	22	34	2.6	1,5	65%	
	total	1049							DMARDs naive	
Quinn et al. (22) Infliximab+MTX vs. MTX J [3]	3 mg/Kg 8 wk *	10	51	0,6	NA	NA	4.7	1,3	Not previously DMARDs	Not previously MTX
	MTX	10	53	0,5			3.7	1.3		
	total	20								
Westhovens et al. (23) Infliximab+MTX vs. MTX J [4]	3 mg/Kg 8 wk *	360	53	7.8	15	22	1.6	1.5	NA	Insufficient
	10 mg/Kg 8 wk *	361	52	6.3	15	22	1.6	1.5		
	MTX	363	52	8.4	15	22	1.2	1.5		
	total	1084								
Moreland et al. (24) Etanercept vs. placebo J [5]	25 mg twice weekly *	78		11	25	33	4.7	1.6	3.3	Insufficient
	10 mg twice weekly	76	53	13	25	34	5.3	1.7	3.4	
	placebo	80	53	12	25	35	4.1	1.7	3.0	
	total	234	51							
Weinblatt et al. (25) Etanercept+MTX vs. MTX J [3]	25 mg twice weekly *	59		13	20	28	2.2	1.5	2.7	Insufficient
	MTX	30		13	17	28	2.6	1.5	2.8	
	total	89	48							
			53							
Bathon et al. (26) Etanercept vs. MTX J [2]	25 mg twice weekly *	207	51	1	24	31	3.3	NA	0.5	Not previously MTX
	10 mg twice weekly	208	50	0.9	24	31	4.4		0.5	
	MTX	217	49	1	24	30	3.7		0.6	
	total	632								
van der Heijde et al. (28) (TEMPO) Etanercept+MTX vs. MTX vs. Etanercept J [4]	25 mg twice weekly +MTX *	231		7	22	34	2.9	NA	2.3	
	25 mg twice weekly *	223		6	23	35	2.2		2.3	
	MTX	228	52	7	22	33	3.5		2.3	
	total	682	53							
			53							
Weinblatt et al. (29) (ARMADA) Adalimumab+MT X vs. MTX J [3]	40 mg/2 wk *	67	57	12	17	28	2.1	1.5	2.9	Insufficient
	20 mg/2 wk	69	53	13	17	28	2.8	1.5	3.0	
	80 mg/2 wk	73	55	12	17	30	2.8	1.5	3.1	
	MTX	62	56	11	16	28	3.1	1.6	3.0	
	total	271								
van de Putte et al. (30) Adalimumab vs. Placebo J [5]	40 mg/2 wk *	113	52	10	20	33	5.2	1.8	3.8	Insufficient
	20 mg/2 wk	106	53	9	19	33	5.2	1.8	3.7	
	20 mg/wk	112	54	11	19	35	4.7	1.8	3.6	
	40 mg/wk	103	51	11	19	33	4.9	1.8	3.8	
	Placebo	110	53	11	19	35	5.7	1.8	3.6	
	total	544								
Furst et al. (31) (STAR) Adalimumab+ DMARD vs. DMARD J [3]	40 mg/2 wk *	318		9	20	27	1.5	1.3	57–60%	Insufficient
	DMARD	318		11	21	27	1.5	1.4	2 or plus	
	total	636	55							
			55							
Keystone et al. (32) Adalimumab+MT X vs. MTX J [3]	40 mg/2 wk *	207	56	11	19	27	1.8	1.4	2.4	Insufficient
	20 mg/wk	212	57	11	19	27	1.4	1.4	2.4	
	MTX	200	56	10	19	28	1.8	1.4	2.4	
	total	619								
Breedveld et al. (33) (PREMIER) Adalimumab+MT X vs. MTX vs. Adalimumab J [4]	40 mg/2 wk + MTX *	268	52	0,7	21	31	NA	1.5	NA	Not previously MTX
	40 mg/2 wk	274	52	0,7	22	32		1.6		
	MTX	257	52	0,8	22	32		1.5		
	total	799								

*groups receiving doses currently recommended

NA: not available

CRP: C-reactive protein

HAQ: health assessment questionnaire

### Trial characteristics

Table 1 shows the major characteristic of the 13 trials included in the review. Information on efficacy at 6, 12 and 24 months and the previously-described safety parameters were analyzed.

There are four infliximab trials: Lipsky et al. [19], St. Clair et al. [20], Quinn et al. [22] and Westhovens et al. [23]. Lipsky et al. [19] used a randomised double blind 12-month trial (with information at 6 and 12 months). Four hundred and twenty-eight patients insufficiently responsive to MTX were included. Patients were randomized to 5 arms, four with infliximab plus MTX and a control arm with MTX alone. The purpose of this study was to demonstrate that infliximab was capable of inhibiting the progression of structural joint damage. It was a continuation of the Maini et al. trial [21]. The St. Clair et al. trial [20] compared infliximab plus MTX with MTX alone. The 1049 patients included in this trial had a recent onset of RA (disease duration in the range 3 months to 3 years). They had not previously received MTX, and MTX was administered following a rapid dose-increasing schedule during the trial (7.5 mg/wk at week 0, increasing by 2.5 mg per week to 15 mg/wk at week 5 and 20 mg/wk at week 8). The trial lasted 12 months. The Quinn et al. [22] trial was a small study comparing infliximab plus MTX with MTX alone in recent-onset RA patients. Westhovens et al. [23] conducted a 12-month trial but the double blind efficacy data were analyzed at week 22.

Four trials testing etanercept were analysed. Moreland et al. [24] compared etanercept in monotherapy with a placebo. Weinblatt et al. [25] compared etanercept plus MTX with MTX alone. In the Bathon et al. trial [26], etanercept in monotherapy was compared with MTX. Finally, the TEMPO trial (Trial of Etanercept and Methotrexate with radiographic Patient Outcomes) [28] compared etanercept in monotherapy with both MTX and a combination of etanercept plus MTX. Moreland et al. included 234 patients [24] in a 6 month-trial comparing the response to etanercept with placebo. Patients had previously shown an inadequate response to at least 1 DMARD (80% to MTX). They had received an average of three DMARDs and were therefore defined as refractory to standard treatments. Weinblatt et al. [25] included 89 patients with inadequate responses to MTX. The duration of the trial was 6 months. Bathon et al. [26] recruited 632 patients with recent RA onset (less than 3 years' duration). Patients ought not to have been treated with MTX previously. The trial lasted 1 year (with information at 6 and 12 months) and MTX was administered in a rapid dose-increasing schedule (initial dose 7.5 mg/wk, increased to 15 mg/wk at week 4 and 20 mg/wk at week 8). The TEMPO trial [28] included 682 patients with RA and insufficient DMARD responses. Around 40% had previously received MTX (but patients previously treated with MTX had neither discontinued it owing to toxicity nor been treated with MTX within 6 months of enrolment). The trial lasted 2 years. MTX was administered in a rapid dose-increasing schedule to 20 mg/wk in 8 weeks. The main goal of this study was to demonstrate that etanercept was capable of inhibiting the progression of structural joint damage.

There were five adalimumab trials. The ARMADA trial (The Anti-tumour necrosis factor Research study programme of the Monoclonal Antibody adalimumab in rheumatoiD Arthritis) [29] compared the efficacy of 6 months' treatment with adalimumab plus MTX with MTX alone in 271 patients with insufficient responses to MTX and at least one other DMARD. The van de Putte et al. trial [30] compared the efficacy of adalimumab with a placebo after 6 months treatment. All 544 patients included had inadequate responses to MTX and 3 other DMARDs. The basic purpose of the STAR trial (Safety Trial of Adalimumab in Rheumatoid arthritis) [31] was to study adalimumab safety. It recruited 636 patients with inadequate responses to any DMARD who were subsequently randomised either to continue with their current DMARD alone or to use a combination of the DMARD with adalimumab for 6 months. The Keystone et al. [32] trial had a similar design to ARMADA with a 12-month duration (information at 6 and 12 months), comparing adalimumab plus MTX with MTX alone in 619 patients. The basic purpose of this study was to demonstrate that adalimumab could inhibit the progression of structural joint damage. The PREMIER trial [33] compared the efficacies of adalimumab, adalimumab plus MTX and MTX alone in 799 patients at 24 months without previous treatment with MTX.

For each selected trial we extracted data on major features of the study design and characteristics of the patients included (Table 1). Weekly doses of MTX administered to the patients during the trials averaged 16 mg, except for the St. Clair et al. [20], TEMPO [28]. Bathon et al. [26] and PREMIER trials [33], in which MTX was administered in a rapid dose-increasing schedule to 20 mg/wk. The clinical profile of RA also varied across trials. Patients had a long history of RA (around 10 years) in most trials but a shorter evolution time in four of them: under 3 years in the St. Clair et al. [20], Bathon et al. [26] and Quinn et al. [22] and PREMIER [33] trials and around 6 years in the TEMPO [28] study. Therapeutic use of MTX prior to enrolment in the trial was also considered, because failure of or inadequate response to prior MTX administration entails a low response rate in the control group.

### Metaanalysis results

#### Efficacy of anti-TNFα drugs. Global analysis

**Figure 2:**
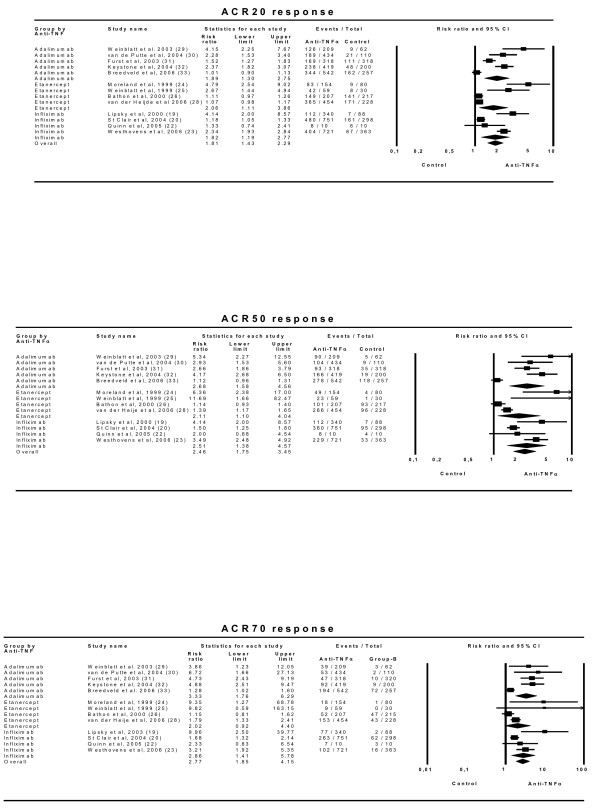
**Efficacy of all doses of anti-TNFα drugs on ACR20, ACR50 and ACR70 responses. **Effect refers to the risk of obtaining the corresponding response with anti-TNFα drug relative to control treatment. 'Lower' and 'upper' represent the 95% confidence interval limits for the efficacy estimate. Random-effect models.

We studied the efficacies of the anti-TNFα drugs in the 13 trials included [19-33] (Table 2). Global comparison of the ACR20 efficacy of any dose of any anti-TNFα drug with any control treatment showed a combined effect of 1.81 (95% CI 1.43–2.29) with an NNT of 6 (5–7). The combined effects were 1.89 (1.30–2.75) for adalimumab trials, 1.71 (1.11–2.63) for etanercept trials and 1.82 (1.19–2.77) for infliximab trials. Further analyses using ACR50 and ACR70 efficacies showed very similar results (Figure 2).

**Table 2:** Efficacy of anti-TNFα drugs on ACR20, ACR50 and ACR70 responses

**Trial (reference) Comparisons Duration of trial in months**	**Groups**	**N of patients**	**6 month ACR20**	**6 month ACR50**	**6 month ACR70**	**12 month ACR20**	**12 month ACR50**	**12 month ACR70**	**24 month ACR20**	**24 month ACR50**	**24 month ACR70**
Lipsky et al. (19) Infliximab+MTX vs. MTX 12 months	3 mg/Kg 8 wk +MTX*	86		22/86	7/86	36/86	18/86	9/86			
	3 mg/Kg 4 wk +MTX	86	43/86	25/86	9/86	41/86	29/86	31/86			
	10 mg/Kg 8 wk +MTX	87	43/86	27/87	15/87	51/87	34/87	22/87			
	10 mg/Kg 4 wk +MTX	81	45/87	21/81	9/81	48/81	31/81	15/81			
	Total Infliximab	340	47/81	95/340	40/340	176/340	112/340	77/340			
	MTX	88	178/340	4/88	0/88	15/88	7/88	2/88			
	Total	428	18/88								
*St. Clair et al. (20) *Infliximab+MTX vs. MTX 12 months	3 mg/Kg 8 wk +MTX*	373		NA	NA	231/373	171/373	123/373			
	6 mg/Kg 8 wk +MTX	378				249/378	189/378	140/378			
	Total Infliximab	751	NA			480/751	360/751	263/751			
	MTX	298				161/298	95/298	62/298			
	Total	1049									
Quinn et al. (22) Infliximab+MTX vs. MTX 12 months	3 mg/Kg 8 wk +MTX*	10				8/10	8/10	7/10			
	MTX	10	NA	NA	NA	6/10	4/10	3/10			
	Total	20									
Westhovens et al. (23) Infliximab+MTX vs. MTX 6 months	3 mg/Kg 8 wk +MTX*	360		110/360	48/360						
	10 mg/Kg 8 wk +MTX	361	199/360	119/361	54/341						
	Total Infliximab	721	205/361	229/721	102/721						
	MTX	363	404/721	33/363	16/363						
	Total	1084	87/363								
oreland et al. (24) Etanercept vs. placebo 6 months	25 mg 2 twice weekly *	78		31/78	11/78						
	10 mg 2 twice weekly	76	46/78	18/76	7/76						
	Total Etanercept	154	37/76	49/154	18/154						
	Placebo	80	83/154	4/80	1/80						
	Total	234	9/80								
Weinblatt et al. (25) Etanercept+MTX vs. MTX 6 months	25 mg 2 +MTX *	59	42/59	23/59	9/59						
	MTX	30	8/30	1/30	0/30						
	Total	89									
Bathon et al. (26) ** *Etanercept vs. MTX *12 months	25 mg 2 twice weekly *	207	147/207	NA	NA	149/207	101/207	52/207			
	10 mg 2 twice weekly	208	NA	NA	NA	NA	NA	NA			
	Total Etanercept	415	56/217	NA	NA	141/217	93/217	47/217			
	MTX	217									
	Total	632									
van der Heijde et al. (28) (TEMPO) Etanercept+MTX vs. etanercept vs MTX 24 months	25 mg 2 twice weekly +MTX *	231	NA	NA	NA	196/231	159/231	99/231	199/231	164/231	113/231
	25 mg 2 twice weekly*	223				169/223	107/223	54/223	167/223	120/223	60/223
	Total Etanercept	454				365/454	266/454	153/454	386/454	284/454	173/454
	MTX	228				171/228	98/228	43/228	162/228	96/228	4/228
	Total	682									
Weinblatt et al. (29) ARMADA) dalimumab+MTX vs. MTX 6 months	40 mg/2 s+MTX*	67	45/67	37/67	18/67						
	20 mg/2 s+MTX	69	33/69	22/69	7/69						
	80 mg/2 s+MTX	73	48/73	31/73	14/73						
	Total Adalimumab	209	126/209	90/209	39/209						
	MTX	62	9/62	5/62	3/62						
	Total	271									
van de Putte et al. (30) Adalimumab vs. Placebo 6 months	40 mg/2 wk *	113		25/113	14/113						
	20 mg/2 wk	106	52/113	20/106	9/106						
	20 mg/wk	112	38/106	23/112	11/112						
	40 mg/wk	103	44/112	36/103	19/103						
	Total Adalimumab	434	55/103	104/434	53/434						
	Placebo	110	189/434	9/110	2/110						
	Total	544	21/110								
Furst et al. (31) (STAR) Adalimumab+DAMAR D vs. DAMARD 6 months	40 mg/2 wk *	318		93/318	47/318						
	DMARD	318	169/318	35/318	10/318						
	Total	636	111/318								
Keystone et al. (32) Adalimumab+MTX vs. MTX 12 months	40 mg/2 wk +MTX*	207	131/207	80/207	43/207	122/207	86/207	48/207			
	20 mg/wk +MTX	212	129/212	87/212	36/212	116/212	80/212	44/212			
	Total Adalimumab	419	260/419	167/419	79/419	238/419	166/419	92/419			
	MTX	200	59/200	19/200	5/200	48/200	19/200	9/200			
	Total	619									
Breedveld et al. (33) (PREMIER) Adalimumab+MTX vs. adalimumab vs MTX 24 months	40 mg/2 wk+MTX*	268	NA	*NA*	NA	196/268	166/268	123/268	185/268	158/268	126/268
	40 mg/2 wk *	274				148/274	112/274	71/274	134/274	101/274	77/274
	Total Adalimumab	542				344/542	278/542	194/542	319/542	259/542	203/542
	MTX	257				162/257	118/257	72/257	144/257	111/257	72/257

* groups receiving doses currently recommended

NA: not available

**Figure 3:**
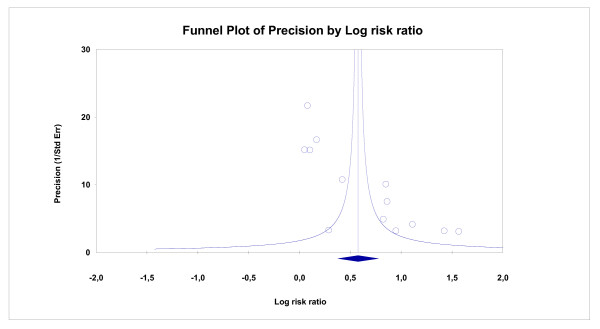
**Funnel plot of selected studies.** The x-axis shows effect estimates (RR) on a logarithmic scale (any effect estimate greater than zero therefore indicates better results for experimental treatment) while the y-axis measures the precision of each study (as the inverse of the standard error of the effect estimate measured on a logarithmic scale).

Analysis of this set of 13 trials provided evidence of relevant and statistically significant heterogeneity (Q = 157.7; p < 0.001; I^2 ^92%). Although the limited number of trials reduced the discriminatory power of the funnel plot, it suggested a certain degree of asymmetry (Figure 3), which was statistically confirmed by both the Begg and Mazumdar adjusted rank correlation test (p = 0.033) and Egger's regression asymmetry test (p = 0.001).

Bearing all these aspects in mind, we focused on the analysis of subgroups that appeared more homogeneous on both clinical and trial design grounds: previous exposure and response to DMARDs, mainly MTX, dose of anti-TNFα drug administered and control treatment selected (active or placebo, single or combined). The effects (RR) obtained with different doses of anti-TNFα appear in Table 3. The distinct NNTs and the analysis of heterogeneity appear in Table 4. Evidently the specific subgroups of trials characterised by these features are much less heterogeneous on analysis.

**Table 3:** Effects (RR and NNT (95% CI)) obtained with different doses of anti-TNFα drugs

		All doses of anti-TNFα drugs vs. control 4618 vs. 2261*		Recommended doses of anti-TNFα drugs vs. control 2874 vs. 2260**		High-doses drugs vs. control 1169 vs. 921*** of anti-TNFα		Low-doses of anti-TNFα drugs vs. control 251 vs. 252****	
Anti-TNFα	ACR	RR (CI 95%)	NNT	RR (CI 95%)	NNT	RR (CI 95%)	NNT	RR (CI 95%)	NNT

Adalimumab	ACR20	1.9 (1.3–2.8)	6 (5–7)	2.0 (1.3–2.9)	5 (4–6)	3.5 (1.6–7.3)	3 (2–4)	2.4 (1.4–4.1)	5 (4–8)
	ACR50	2.7 (1.6–4.4)	6 (5–7)	2.8 (1.6–4.7)	5 (5–6)	4.7 (1.9–12.0)	4 (3–5)	2.9 (1.6–5.1)	7 (5–13)
	ACR70	3.3 (1.8–6.3)	9 (7–11)	3.5 (1.9–6.7)	7 (6–8)	6.1 (1.8–20.8)	7 (5–11)	3.0 (1.1–7.9)	17 (9–77)
Etanercept	ACR20	1.7 (1.1–2.6)	7(5–10)	1.7 (1.1–2.7)	6 (5–8)	There are no trials with high doses of Etanercept		4.3 (1.9–10.1)	3 (2–5)
	ACR50	2.1 (1.1–3.9)	6 (5–9)	2.2 (1.1–4.3)	6 (4–7)			4.7 (1.7–13.4)	6 (4–13)
	ACR70	2.0 (0.9–4.4)	NS	2.1 (0.9–4.5)	NS			7.4 (0.9–58.5)	NS
Infliximab	ACR20	1.8 (1.2–2.8)	5 (4–6)	1.7 (1.1–2.6)	5 (4–6)	2.0 (1.2–3.6)	5 (4–5)	There are no trials with low doses of Infliximab	
	ACR50	2.6 (1.5–4.7)	5 (5–6)	2.2 (1.2–4.1)	6 (5–7)	2.8 (1.5–5.5)	5 (4–6)		
	ACR70	2.9 (1.4–5.8)	8 (6–10)	2.4 (1.2–5.0)	9 (7–13)	3.3 (1.5–7.2)	7 (6–7)		
Overall	ACR20	1.8 (1.4–2.3)	6 (5–7)	1.8 (1.4–2.3)	5 (5–6)	2.5 (1.5–4.2)	4 (5–4)	2.9 (1.7–5.1)	4 (3–6)
	ACR50	2.5 (1.8–3.4)	6 (5–6)	2.4 (1.7–3.4)	5 (5–6)	3.4 (2.0–5.8)	5 (4–5)	3.2 (2.0–5.3)	6 (5–10)
	ACR70	2.8 (1.9–4.2)	8 (7–9)	2.7 (1.8–4.1)	7 (7–9)	3.9 (2.0–7.6)	7 (6–8)	3.5 (1.4–8.6)	15 (10–38)

NNT: number of patients needed to be treated

RR (95%CI): relative risk (95% confidence limits)

NS: non-significant results

*4618 patients being treated with anti-TNFα (except 208 patients Bathon's trial being treated with 10 mg of etanercept twice a week) vs 2261 patients of the control groups

**2874 patients with recommended doses of anti-TNFα drugs (Infliximab 3 mg/Kg/8 week; etanercept 25 mg twice a week; adalimumab 40 mg every 2 weeks) vs 2260 patients of the control groups

***1169 patients with high-doses of anti-TNFα drugs (infliximab 3 mg/Kg 4 week, 6 mg/Kg 8 week, 10 mg/Kg 8 week and 10 mg/Kg 4 week; adalimumab 40 mg/week, 80 mg/2 week) vs 921 patients of the control groups

****251 patients with low-doses of anti-TNFα drugs (etanercept 10 mg twice weekly; adalimumab 20 mg/2 week) vs 252 patients of the control groups

**Table 4:** Efficacy and heterogeneity

Comparisons (Anti-TNFα vs. control)***	**ACR response**	**Anti-TNFα Events/Total**	**Control Events/Total**	**RR (CI 95%)**	**NNT**	**Q**	**I**^2^%
All doses of anti-TNFα drugs vs. control (4618 vs. 2261)	ACR20	2709/4618	941/2261	1.8 (1.4–2.3)	6 (5–7)	157.7*	92
	ACR50	1879/4618	519/2261	2.5 (1.8–3.4)	6 (5–6)	109.8*	89
	ACR70	1106/4618		2.8 (1.9–4.1)	8 (7–9)	52.4*	77
Recommended doses of anti-TNFα drugs vs. control (2874 vs. 2260)	ACR20	1808/2874	949/2261	1.8 (1.4–2.3)	5 (5–6)	149.5*	92
	ACR50	1247/2874	519/2261	2.4 (1.7–3.4)	5 (5–6)	102.9*	88
	ACR70	733/2874	270/2261	2.7 (1.8–4.1)	7 (7–9)	49.2*	76
High-doses of anti-TNFα drugs vs. control (1169 vs. 921)	ACR20	697/1169	293/921	2.5 (1.5–4.2)	4 (4–5)	57.2*	93
	ACR50	469/1169	149/921	3.4 (2.0–5.8)	5 (4–5)	30.5*	87
	ACR70	295/1169	85/921	3.9 (2.0–7.6)	7 (6–8)	16.6*	76
*Low-doses of anti-TNFα drugs vs. control (251 vs. 252)*	ACR20	108/251	39/252	2.9 (1.7–5.1)	4 (3–6)	4.9	59
	ACR50	60/251	18/252	3.2 (2.0–5.3)	6 (5–10)	1.5	0
	ACR70	23/251	6/252	3.5 (1.4–8.6)	15 (10–38)	1.2	0
Anti-TNFα drugs vs. control in patients with No insufficient response to MTX (1964 vs. 1010)	ACR20	1346/1964	641/1010	1.1 (0.9–1.3)	NS	2.4	9
	ACR50	1013/1964	408/1010	1.3 (1.1–1.5)	9 (7–13)	8.8	55
	ACR70	669/1964	227/1010	1.5 (1.3–1.7)	12 (9–19)	7.0	43
Anti-TNFα drugs vs. control in patients with insufficient response to MTX (2654 vs. 1251)	ACR20	1427/2654	308/1251	2.3 (2.0–2.7)	4 (3–5)	28.2*	75
	ACR50	866/2654	113/1251	3.6 (2.9–4.4)	5 (4–5)	7.2	3
	ACR70	437/2654	43/1251	4.4 (3.2–6.0)	7 (6–8)	4.2	0
Anti-TNFα drugs at recommended doses plus MTX vs. MTX alone in patients with insufficient response to MTX (779 vs. 743)	ACR20	444/779	167/743	2.6 (2.1–3.3)	3 (3–4)	4.3	7
	ACR50	274/779	65/743	4.1 (2.6–6.6)	4 (4–5)	4.6	13
	ACR70	132/779	30/743	4.1 (2.4–7.1)	8 (7–11)	2.5	0
Anti-TNFα drugs versus placebo at recommended doses (191 vs. 190)	ACR20	98/191	30/190	3.4 (1.6–7.3)	3 (3–4)	3.8*	74
	ACR50	56/191	13/190	4.4 (1.5–12.5)	5 (4–7)	2.9	66
	ACR70	25/191	3/190	8.1 (2.5–26.4)	9 (7–16)	0.1	0
Anti-TNFα drugs at recommended doses plus MTX versus MTX alone in patients with no previous resistance to MTX (882 vs. 793)	ACR20	631/882	500/793	1.2 (1.1–1.2)	10 (7–16)	0.4	0
	ACR50	504/882	315/793	1.6 (1.4–1.7)	6 (5–8)	1.1	0
	ACR70	352/882	180/793	1.8 (1.5–2.1)	6 (5–8)	3.9	23
Anti-TNFα drugs versus MTX at recommended doses (704 vs. 702)	ACR20	466/704	474/702	1.0 (0.9–1.1)	NS	6.9*	71
	ACR50	320/704	309/702	1.0 (0.9–1.2)	NS	3.6	45
	ACR70	177/704	162/702	1.1 (0.9–1.3)	NS	2.2	11

RR (95%CI): relative risk (95% confidence limits)

NNT: number of patients needed to be treated

Q: Cochrane's Q

I^2^: percentage of variability in study results attributable to between-study differences

* statistical heterogeneity

NS: non-significant results

*** number of patients being treated with anti-TNFα versus number of patients in the control groups

#### Efficacy of anti-TNFα drugs depending on prior exposure and response to MTX

**Figure 4:**
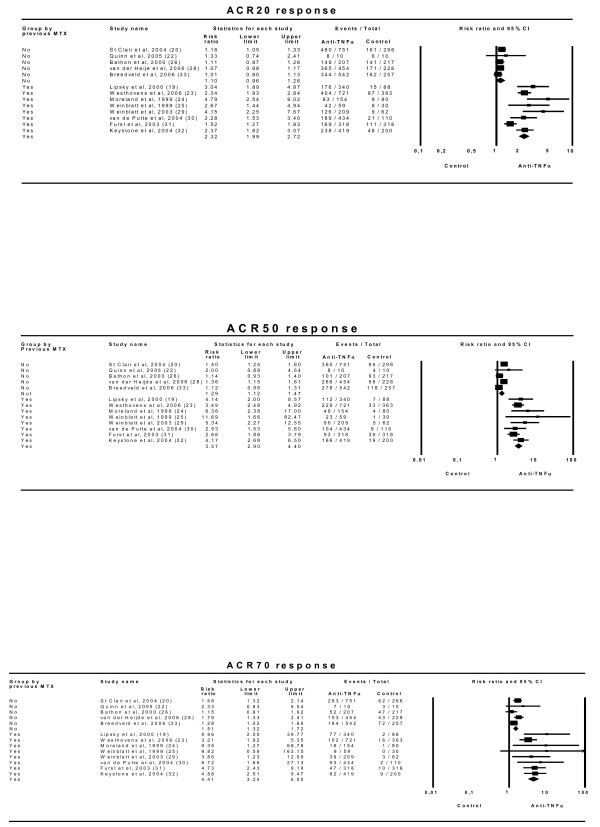
**Efficacy of anti-TNFα drugs depending on an insufficient response to MTX prior to trial commencement.** Effect refers to risk of obtaining the corresponding response with anti-TNFα drug relative to control treatment. 'Lower' and 'Upper' represent the 95% confidence interval limits for the efficacy estimate. Random-effect models.

The efficacy results (Figure 4) show that this factor leads to rather different estimates and should be taken into account. When the effect of an anti-TNFα drug is assessed in patients who have received no previous MTX treatment, the relative ACR20 effect is small and only marginally statistically significant: 1.10 (0.96–1.26). On the other hand, when the anti-TNFα drug effect is analysed in patients with previously insufficient responses to MTX, the relative effect is substantially larger (2.32 [1.99–2.72]) and both clinically relevant and statistically significant [NNT of 4 (3–5)]. Similar results are seen with the ACR50 and ACR70 responses, though here the effect in patients naïve to MTX is statistically significant compared to control arms.

#### Analysis of the effect of different doses of anti-TNFα drugs

We analysed the efficacy of anti-TNFα drug administration in three separate groups: currently recommended doses (infliximab 3 mg/Kg/8 week; etanercept 25 mg twice a week; adalimumab 40 mg every 2 weeks); high doses (infliximab 3 mg/Kg/4 week, 6 mg/Kg/8 week, 10 mg/Kg/8 week and 10 mg/Kg/4 week; adalimumab 40 mg/week, 80 mg/2 week); and low doses (etanercept 10 mg 10 mg twice weekly; adalimumab 20 mg/2 week). No patient receiving infliximab was prescribed lower than recommended doses, and no patient treated with etanercept received higher than recommended doses. The group given adalimumab 20 mg/week was not included as this dose schedule can be considered neither above nor below the currently recommended regime. The combined and individual effects of the adalimumab, etanercept and infliximab trials at any dose or in subgroups based on the dose level are shown in Table 3. A statistically significantly beneficial effect is apparent with recommended, higher or lower doses in all the comparisons made, except for the ACR70 response to etanercept. Accordingly, the NNTs are very similar for all anti-TNFα drugs.

#### Analysis of the effect of anti-TNFα drugs at recommended doses

**Figure 5:**
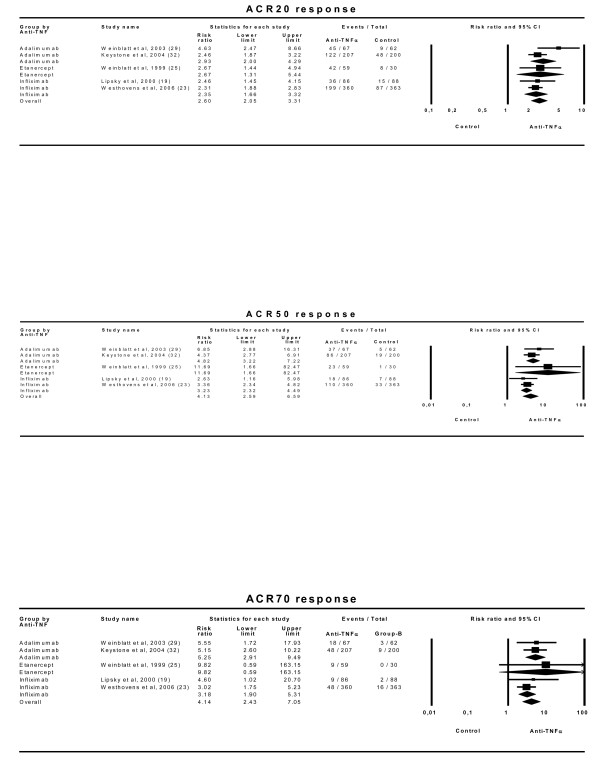
**Efficacy of anti-TNFα drugs at recommended doses in combination with MTX compared with MTX alone in patients with insufficient responses to MTX.** Effect refers to risk of obtaining the corresponding response with anti-TNFα drug relative to control treatment. 'Lower' and 'Upper' represent the 95% confidence interval limits for the efficacy estimate. Random-effect models.

Five trials [19,23,25,29,32] compared the effects of anti-TNFα drugs plus MTX with MTX alone in patients with insufficient responses to MTX. A beneficial combined effect in the ACR20 response is shown: RR 2.60 (2.05–3.31) with an NNT of 4 (3–4). Analyses using the ACR50 and ACR70 responses showed very similar results (Figure 5). There was no evidence of statistical heterogeneity among the different drug classes (Table 4).

**Figure 6:**
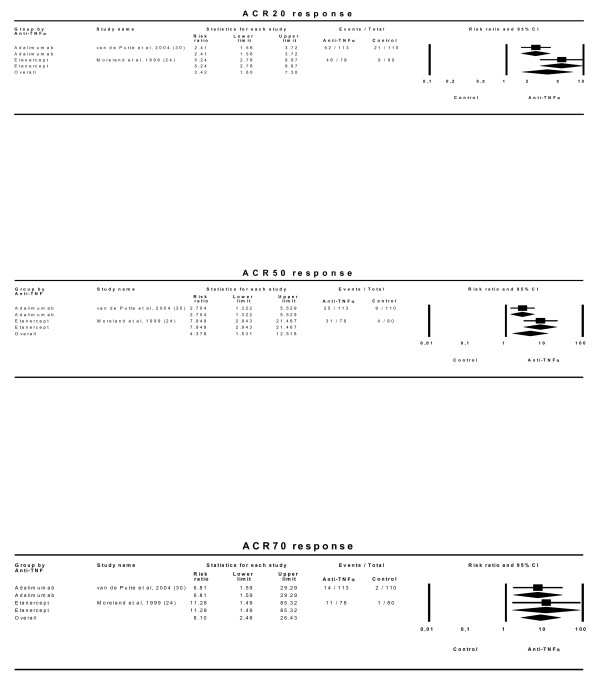
**Efficacy of anti-TNFα drugs versus placebo at recommended doses.** Effect refers to risk of obtaining the corresponding response with anti-TNFα drug relative to control treatment. 'Lower' and 'Upper' represent the 95% confidence interval limits for the efficacy estimate. Random-effect models.

Only two trials [24,30] assessed the effect of anti-TNFα drugs versus placebo, showing a combined positive effect on the ACR20 response with an RR of 3.42 (1.60–7.30) and an NNT of 3 (3–4). Although there was a statistically significant heterogeneity of effects according to which drug had been used (Q = 3.8; p = 0.049; I^2 ^= 74) with etanercept apparently more effective than adalimumab (Figure 6), it should be emphasized that there was no direct head to head comparison among them. There was no statistical evidence of heterogeneity in either the ACR50 or the ACR70 response, but the estimates of the effect varied widely between the two drugs, with a pattern similar to that obtained with the ACR20 outcome and based on a rather small number of patients.

**Figure 7:**
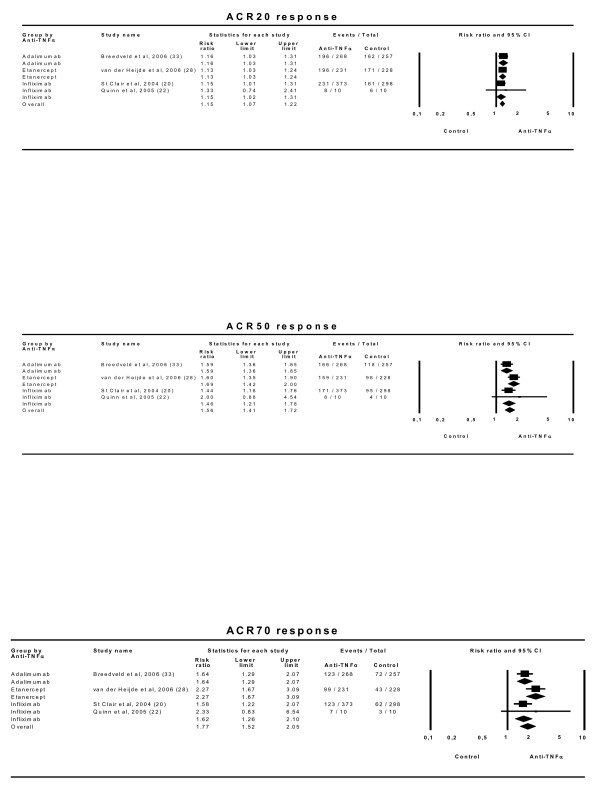
**Efficacy of anti-TNFα drugs plus MTX compared to MTX alone in patients with no previous resistance to MTX (at the recommended doses).** Effect refers to risk of obtaining the corresponding response with anti-TNFα drug relative to control treatment. 'Lower' and 'Upper' represent the 95% confidence interval limits for the efficacy estimate. Random-effect models.

Four trials [20,22,28,33] compared the effect of anti-TNFα drug plus MTX with MTX alone in patients with no previous resistance to MTX. This analysis showed a small but significant combined effect on the ACR20 response of 1.15 (1.07–1.22) with an NNT of 10 (7–16) (Figure 7). The ACR50 showed a combined effect of 1.56 (1.41–1.72) whereas that effect was 1.77 (1.52–2.05) for ACR70. No statistically significant heterogeneity was present for any of these outcomes.

**Figure 8:**
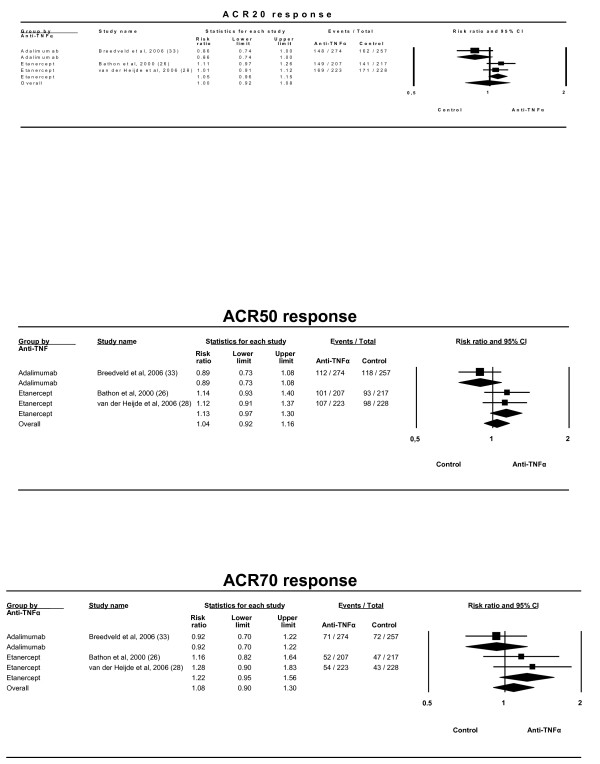
**Efficacy of anti-TNFα drugs compared to MTX at recommended doses.** Effect refers to risk of obtaining the corresponding response with anti-TNFα drug relative to control treatment. 'Lower' and 'Upper' represent the 95% confidence interval limits for the efficacy estimate. Random-effect models.

Three trials compared efficacies of anti-TNFα drugs with MTX alone as control [26,28,33]. The ACR20 combined effect showed no significant difference among the arms, with RR = 1.00 (0.92–1.08). Results were similar for the ACR50 and ACR70 responses (Figure 8). The heterogeneity was marginally significant for ACR20 and significant for ACR50 (Table 4).

### Safety analysis

An overview of the adverse events reported in the selected trials is displayed in Table 5. The number of withdrawals due to adverse events according to treatment arm was reported in all trials. Information on the incidence of serious infections, malignancies and mortality is also provided, specifying whether patients were in the experimental or control arms, but information about the specific treatment group of the patient was sometimes lacking. Other important safety information (overall number of adverse events, severe adverse events, total number of infections, infusion reactions and injection-site reactions) was provided much less consistently.

**Table 5:** Number of patients who presented adverse effects in trials with anti TNFα drugs

**Trial (reference) Anti-TNFα drug**	**Groups**	**N of patients**	**Withdrawn adverse event**	**Total adverse events**	**Serious adverse events**	**Infections**	**Serious infections**	**Infusion reactions**	**Injection- site reactions**	**Malignancies**	**Mortality**
Lipsky et al. (19) Infliximab	3 mg/Kg 8	86	5	_	_	_	_			_	_
	wk +MTX	86	9	_	_	_	_			_	_
	3 mg/Kg 4	87	4	_	_	_	_			_	_
	wk +MTX	81	8	_	_	_	_			_	_
	10 mg/Kg 8	340	26	323	58	244	22			5	5
	wk +MTX	88	7	83	18	53	7	NA		0	3
	10 mg/Kg 4										
	wk +MTX										
	Total										
	Infliximab										
	MTX										
St. Clair et al. (20) Infliximab	3 mg/Kg 8	373	34		_	_	21	_		0	1
	wk +MTX	378	35		_	_	19	_		4	1
	6 mg/Kg 8	751	69		103	414	40	135		4	2
	wk +MTX	298	9	NA	32	141	6	20		0	2
	Total										
	Infliximab										
	MTX										
Quinn et al. (22) Infliximab	3 mg/Kg 8	10	1		1		0	1		0	0
	wk +MTX	10	0	NA	0	NA	0	0		0	0
	MTX										
Westhovens et al. (23) Infliximab	3 mg/Kg 8	360	18	_	_		6			2	0
	wk +MTX	361	20	_	_		18			2	2
	10 mg/Kg 8	721	38	512	55		24			4	2
	wk +MTX	363	8	239	27	NA	6	NA		1	1
	Total										
	Infliximab										
	MTX										
Moreland et al. (24) Etanercept	25 mg twice	78	2				0		_	0	0
	weekly	76	5				0		_	0	0
	10 mg twice	154	7				0		71	0	0
	weekly	80	3	NA	NA	NA	0		10	0	0
	Total										
	Etanercept										
	Placebo										
Weinblatt et al. (25) Etanercept	25 mg twice	59	2			30	0		23	0	0
	weekly	30	1	NA	NA	19	0		2	0	0
	+MTX MTX										
Bathon et al. (26) Etanercept	25 mg twice	207	11						_	3	1
	weekly	208	12						_	2	1
	10 mg twice	415	23						140	5	2
	weekly	217	24	NA	NA	NA	NA		16	2	0
	Total										
	Etanercept										
	MTX										
van der Heijde et al. (28) (TEMPO) Etanercept	25 mg twice	231	37	_	_	_	23			5	1
	weekly	223	34	_	_	_	24			5	1
	+MTX	454	71	379	64	285	47		69	10	2
	25 mg twice	228	47	185	37	147	25		4	2	1
	weekly										
	Total										
	Etanercept										
	MTX										
Weinblatt et al. (29) (ARMADA) Adalimumab	40 mg/2 wk	67	0				_		_	_	_
	+MTX	69	4				_		_	_	_
	20 mg/2 wk	73	1				_		_	_	_
	+MTX	209	5				2		32	1	0
	80 mg/2 wk	62	2	NA	NA	NA	0		2	0	0
	+MTX										
	Total										
	Adalimumab										
	MTX										
van de Putte et al. (30) Adalimumab	40 mg/2 wk	113	7	_	_		_		_	_	1
	20 mg/2 wk	106	4	_	_		_		_	_	0
	20 mg/wk	112	3	_	_		_		_	_	0
	40 mg/wk	103	3								0
	Total	434	17	429	53		10		46	4	3
	Adalimumab	110	2	105	16	NA	0		1	1	1
	Placebo										
Furst et al. (31) (STAR) Adalimumab	40 mg/2 wk	318	9	275	17	166	4		62	1	1
	DMARD	318	8	275	22	157	6		37	0	1
Keystone y cols. (32) Adalimumab	40 mg/2 wk	207	26	_	_	_	11		_	_	2
	+MTX*	212	16	_	_	_	5		_	_	1
	20 mg/wk	419	42	391	97	269	16		101	4	3
	+MTX	200	13	181	37	111	1		48	0	0
	Total										
	Adalimumab										
	MTX										
Breedveld et al. (33) (PREMIER) Adalimumab	40 mg/2 wk	268	32	_			9			2	1
	+MTX*	274	26	_			3			4	4
	40 mg/2 wk	542	58	524			12			6	5
	Total	257	19	245	NA	NA	7		NA	4	1
	Adalimumab										
	MTX										

NA: not available

* Overall data provided although specific data per arm not provided

**Figure 9:**
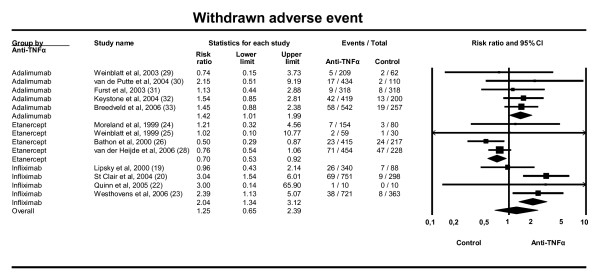
Adverse event withdrawn in patients with all doses of anti-TNFα drugs.

Regarding withdrawals due to adverse events, we found no significant overall difference between the experimental and control groups, with a pooled RR of 1.25 (0.65–2.39) (Figure 9). Results differed depending on the specific anti-TNFα given: patients in the etanercept arms were less likely to withdraw from adverse events than their control counterparts, but the opposite was the case for adalimumab and infliximab, all those comparisons reaching statistical significance. There was statistically significant heterogeneity among the drugs (Q = 29.3; p = 0.003; I^2 ^59) but not within the groups given each specific drug. The results were the same when only groups receiving recommended doses of anti-TNFα drugs were studied. Higher than recommended doses of infliximab led to a higher withdrawal rates. There were no significant differences in withdrawal rates between lower than recommended dose and control arms.

There were more adverse events in patients allocated to anti-TNFα drugs (RR 1.02 (1.00–1.04)) (p = 0.021) (Table 6). Patients receiving infliximab showed a higher frequency of serious adverse events (p = 0.048) and infections (p = 0.004), but the combined estimates for all three anti-TNFα drugs and safety outcomes were not significant.

**Table 6:** Adverse events in patients being treated with anti-TNFα drugs versus control

ADVERSE EVENTS (anti TNFα vs. control) (references)	Anti-TNFα	Anti-TNFα Adverse events/total	Controls Adverse events/total	RR (95%CI)	NNH(95%CI)	Q	I^2 ^%
Withdrawn adverse event	Adalimumab	131/1922	44/947	1.4(1.0–2.0)	47(26–251)	1.2	0
(4826 vs. 2261)	Etanercept	103/1082	75/555	0.7(0.5–0.9)	-26(-143 a -14)	2.4	0
** (19,20,22,23,24,25,26,28,29,30,31,32,33)	Infliximab	134/1822	24/759	2.0(1.3–3.1)	24(17–41)	4.9	0
	Total	368/4826	143/2261	1.3(0.7–2.4)	NS	29.3*	59
Total adverse events (3228 vs. 1564) (19,23,28,30,31,32,33)	Adalimumab	1619/1713	806/885	1.1(0.9–1.1)	NS	1.9	0
(3228 vs. 1564)	Etanercept	379/454	185/228	1.0(0.9–1.1)	NS	0	0
(19,23,28,30,31,32,33)	Infliximab	835/1061	322/45	1.0(0.9–1.0)	NS	1.6	39
	Total	2833/3228	1313/1564	1.0(1.0–1.5)	27(17–59)	2.9	0
Serious adverse events	Adalimumab	167/1171	75/628	1.0(0.7–1.4)	NS	2.6	25
(3235 vs. 1615)	Etanercept	64/454	37/228	0.9(0.5–1.6)	NS	0	0
(19,20,22,23,28,30,31,32)	Infliximab	217/1610	77/759	1.4(1.0–2.0)	31(17–167)	6.2	52
	Total	448/3235	189/1615	1.1(0.8–1.6)	NS	14.3*	51
Infections	Adalimumab	435/737	268/518	1.1(0.9–1.2)	NS	0.7	0
(2341 vs. 1162)	Etanercept	315/513	166/258	1.0(0.9–1.0)	NS	0.9	0
(19,20,25,28,31,32,33)	Infliximab	658/1091	194/386	1.2(1.1–1.3)	10(7–24)	0.03	0
	Total	1408/2341	628/1162	1.9(0.9–1.2)	NS	8.6	41
Serious infections	Adalimumab	44/1922	14/947	1.2(0.6–2.8)	NS	5.8	31
(4188 vs.1937)	Etanercept	47/454	25/28	0.9(0.4–2.3)	NS	0	0
(19,20,22,23,24,25,28,29,30,31,32,33)	Infliximab	90/1812	19/726	1.8(0.9–3.4)	NS	2.7	26
	Total	181/4188	58/1937	1.4(0.8–2.2)	NS	11.8	32
Infusión reactions (761 vs. 308) (20,22)	Infliximab	136/761	20/308	2.7(1.7–4.2)	9(7–14)	0.005	0
Injection-site reactions	Adalimumab	241/1380	88/690	1.7(1.0–3.0)	22(13–67)	12.6*	72
(2454 vs. 1245)	Etanercept	303/1074	32/555	5.1(2.9–8.8)	5(4–6)	2.3	0
(24,25,26,28,29,30,31,32)	Total	544/2454	120/1245	3.0(1.0–8.6)	8(7–10)	51.8*	86
Malignancies	Adalimumab	16/1922	5/947	1.1(0.4–2.7)	NS	1.6	0
(4826 vs. 2261)	Etanercept	15/1082	4/555	1.9(0.6–5.7)	NS	0.3	0
(19,20,22,23,24,25,26,28,29,30,31,32,33)	Infliximab	13/1822	1/759	2.6(0.6–11.6)	NS	1.1	0
	Total	44/4826	10/2261	1.5(0.8–3.0)	NS	3.3	0
Mortality	Adalimumab	10/1922	3/947	1.3(0.4–4.7)	NS	2.0	0
(4826 vs. 2261)	Etanercept	4/1082	1/555	1.5(0.2–9.5)	NS	0.2	0
(19,20,22,23,24,25,26,28,29,30,31,32,33)	Infliximab	9/1822	5/759	0.5(0.2–1.4)	NS	0.4	0
	Total	23/4826	9/2261	0.8(0.3–2.1)	NS	4.4	0

RR (95%CI): relative risk (95% confidence limits)

NNH: number needed to harm

Q: Cochrane's Q

I^2^: percentage of variability in study results attributable to between-study differences

* statistical heterogeneity

NS: non-significant results

** this figure include 208 patients of the Bathon trial not included in efficacy studies as efficacy date were not reported

Information on severe infections, malignancies and deaths was provided in all trials except for severe infections in the Bathon trial. No significant combined increases in risk were seen for any of these results.

We also inquired whether higher than recommended doses are associated with higher incidences of adverse events. The reported data were incomplete, however, as the Lipsky et al. trial [19] did not assign the 22 severe infections detected to each corresponding infliximab dose arm. The risk of severe infection when receiving high doses of infliximab [20,23] was significantly increased (p = 0.006) with an NNH of 40 (26–91), but the risk of developing malignancies was not increased (p = 0.116). Nor did the two trials [29,30] administering high doses of adalimumab report the dose received by patients experiencing severe infections.

## Discussion

This study approached a problem of major clinical and socio-economic importance: the efficacy and safety of anti-TNFα drugs in the treatment of rheumatoid arthritis (RA). We have considered these drugs both individually and as a specific therapeutic group. We have evaluated their efficacy as monotherapy and in combination with MTX. In addition, the efficacies of different doses and the safety of these drugs were explored.

Our search of the literature on the efficacy of anti-TNFα drugs in RA identified thirteen clinical trials fulfilling the required criteria for inclusion in the systematic review and metaanalysis. All thirteen were randomised-controlled trials with a minimal follow-up time of 6 months and used comparable standardised parameters of efficacy. Although the general quality of the trials was high, some difficulties became apparent during the review. The number of trials fulfilling the required criteria was small. Furthermore, there were several sources of clinically relevant heterogeneity: different control treatments were used, populations were not homogeneous, follow-up times differed among trials and the doses administered varied widely (Table 1). Also, the funnel plot asymmetry might indicate publication bias or other types of problems.

In our study, combined analysis of the results from all trials using the recommended doses led us to conclude that anti-TNFα drugs (considered as a therapeutic group) show an effect significantly superior to that of control treatments. However, the heterogeneity was very high, calling for subgroups and more homogeneous comparisons. We only evaluated those trials for which relevant homogeneous comparisons were possible, and a substantial reduction in heterogeneity was apparent when we focused on these groups (Table 4). Comparison of the three anti-TNFα drug plus MTX with MTX alone in patients with insufficient responses to MTX showed no significant heterogeneity of effects, yet despite the absence of head to head comparisons we found no evidence whatsoever that the relative effects of individual drugs are different. Etanercept seemed superior to adalimumab when both drugs were compared to placebo. However, the response observed in the control group of the adalimumab study was substantially higher than that of the etanercept reference group, which casts doubts on the actual comparability of the results and makes it difficult to draw definitive conclusions until the drugs have been compared directly in well designed, head to head randomised trials. Anti-TNFα drug plus MTX had a greater effect than MTX alone in patients with no previous resistance to MTX, but the magnitude of this effect was markedly lower than that obtained in patients with previously inadequate responses to MTX. Trials that assessed this specific efficacy issue recruited patients with short-lasting, less severe disease showing high responses to both experimental and control treatments, thus explaining the lower relative and absolute efficacy estimates (Table 4). In fact, the effects achieved with etanercept and adalimumab in these patients were equivalent to those obtained using MTX for the first time.

When the potential influence on efficacy of doses administered was evaluated, both higher and lower doses than are currently recommended seemed to elicit similar effects, except for the effect of lower doses on ACR70. However, comparisons in this last case are based on a small number of patients.

In the light of these findings it seems sensible to advise that current treatment of moderate and severe RA should be started with MTX. Anti-TNFα drugs should be restricted to patients who do not respond sufficiently to DMARD combinations until experimental evidence demonstrates that the new biological drugs have greater efficacy in earlier stages of RA. It might also be potentially useful to start the indicated treatment with a low dose and then increase it as a function of the magnitude of the response. An alternative option might be to start with the current recommended doses and try to decrease them after a significant stable effect is reached, in order to minimise adverse effects. This issue encompasses important clinical and economic implications probably meriting further research.

For a correct interpretation of our results, the fact that our analyses were based on the ACR response should be taken into account. In recent years, another multidimensional index, the DAS index, has been increasingly used [65]. However, it was not used in any of the trials included in the current review. ACR20, 50 and 70 responses are well-known validated response criteria and they were available in all these anti-TNFα studies, enabling us to conduct a combined analysis and statistical evaluation of the results. Another important subject in the evaluation of the response of RA to anti-TNFα drugs is the quantification of radiological damage (inhibition of progression of structural joint damage). The modified Sharp score was analysed in six trials providing 12-month results and showing the ability of infliximab, etanercept and adalimumab to inhibit the progression of structural joint damage in RA [19,20,26,28,32,33]. Nevertheless, several factors deterred us from using this score as an outcome variable: since it is not normally distributed, the way this index was summarised and displayed in the identified trials did not permit statistical pooling of the results. Moreover the clinical implications of this radiological finding are not yet well understood.

Safety issues are also of central concern. Although we focused solely on published results from well-designed randomised controlled trials, our review shows that patients receiving anti-TNFα drugs are more prone to experience adverse events. Although some of the relative safety estimates are statistically significant, their magnitude is rather small and their clinical relevance should be also addressed. Patients on infliximab and adalimumab withdrew from the trial because of adverse events more frequently than patients on etanercept. Treatment with infliximab is associated with higher frequencies of serious adverse events and infections. If high doses are administered, there is also an increased likelihood of severe infections. All in all, the safety/efficacy relationship as estimated by the NNH/NNT ratio appears to be favourable.

Two metaanalysis have been performed previously [66,67] focusing the problem, although none has been published. Both showed a greater efficacy of etanercept against infliximab. A comprehensive technical report addressing these issues, including an economic evaluation, has recently been published by Chen et al. [68]. Although the deadline for inclusion of studies was February 2005, that article pooled information from 29 studies as its inclusion criteria were much broader: it included studies of shorter duration [46,43,46-48,44], studies using other than the recommended routes of administration [49,51,52], studies in which no arm received recommended doses [62] and a trial in which efficacy was not measured using ACR criteria [58]. It also included unpublished studies. Despite this less restrictive approach, the Chen et al. paper likewise confirms the efficacy of all three marketed anti-TNFα drugs at recommended doses, especially when administered to patients with previous resistance to MTX.

A metaanalysis recently published by Bongartz et al. [7] focused on safety matters regarding infliximab and adalimumab. The risk of malignancies and infections was increased when higher doses were administered. There have been some controversies surrounding its conclusions, involving the accuracy of clinical trials with short follow-up as a means of detecting severe adverse events [69]. With respect to severe infections, our metaanalysis, although it detected a higher frequency in the anti-TNFα arms, showed no significant difference. We pooled safety data from the three available treatments whereas Bongartz et al. [7] only analysed infliximab and adalimumab using a fixed effects pooling method. If we restrict our analysis to infliximab and adalimumab and use a fixed effects model, we also find a significantly higher frequency of severe infections (p = 0.047) with an NNH of 61 (41–126). Therefore, it is likely that the use of both drugs, especially in higher than recommended doses, may increase the risk of severe infections. This risk has not so far been shown for etanercept, but as far as we are aware no study with higher than recommended doses has been published. Our results regarding the incidence of malignancies do not agree with those of Bongartz et al. [7], but they also include malignancies developed at a later stage when the trials are no longer underway.

Recently, two systematic reviews with meta-analysis have been published addressing the role of anti-TNFα drugs as added to MTX vs. MTX alone [70,71]. Both articles select a very limited number of trials, and share an important design limitation, namely, they compare clinical trials that recruit both MTX-naïve patients and MTX-resistant patients, which, from our standpoint leads to their results facing validity problems.

There are limitations to our study that are also shared by other published metaanalyses [7,68] and deserve further comment. The number of published studies is scarce, there is significant heterogeneity in some relevant aspects (patient clinical profiles, comparisons undertaken and lengths of follow-up) and information on safety parameters varies widely among trials. We have attempted to deal with these limitations in the original research by designing and applying rather stringent selection criteria so that our results are based on solid coherent evidence. This reinforcement of internal validity might have been at the expense of some loss of generalizability, yet the quality of information excluded is at least controversial. We have provided these pooled NNTs as a kind of effect estimate for average risk patients. In an attempt to minimise the presence of factors known to influence risks and therefore NNTs, we have selected rather homogeneous studies in terms of minimum follow-up, diagnostic criteria and have further made subgroup analyses accounting for several important clinical characteristics. We have performed an extensive and detailed analysis of available efficacy and safety data.

## Conclusion

It may be concluded that the three marketed anti-TNFα drugs are more effective than the corresponding control treatments (MTX or placebo) in RA patients, with an NNT of 5 for ACR20 and ACR50 and of 7 for ACR70 at currently recommended doses (Table 3). High heterogeneity among trials is apparent in key design aspects and is reflected in the results of the combined analysis of all trials, calling for more in-depth assessment of more homogeneous subgroups. When this task is addressed, patients with previously inadequate responses to MTX show similar positive responses when any of the anti-TNFα drugs are added to their treatment regimes. However, when anti-TNFα drug plus MTX is compared with MTX alone in patients with no previous resistance to MTX, the relative efficacy of the combined regime is much lower. Etanercept and adalimumab are superior to the placebo but their effect in monotherapy is similar to that obtained with MTX. Therefore, we advise against starting treatment with anti-TNFα drugs until a lack of adequate response to MTX is clearly documented. Increasing doses lead to no increase in efficacy (Table 3). Analysis of the effect of low anti-TNFα doses suggests that patients treated with etanercept or adalimumab might obtain clinically substantial benefits with doses lower than those currently recommended if indicated on the basis of safety or other grounds.

Overall, patients on anti-TNFα drugs experience adverse events more frequently and those using infliximab and adalimumab have higher withdrawal rates. Infliximab use is associated with a higher likelihood of severe adverse events including severe infections. Interestingly, though, patients using etanercept seem to do so with lower frequency, although this finding might be due to the limitation of the range of doses used to those recommended by the manufacturer. We found no significant difference in the development of malignancies during the follow-up times in the studies. The safety/efficacy relationship is favourable, especially if recommended doses are used. The safety profile of etanercept might be apparently superior because the other drugs were tested over a wider range of doses, including higher than recommended ones.

Although more research is warranted, especially well-powered head to head randomised comparisons of anti-TNFα drugs, our study helps to clarify some frequently encountered questions in the clinical care of RA patients.

## Competing interests

The author(s) declare that they have no competing interests.

## Authors' contributions

AA-R conceived and designed the study and was involved in data extraction, analysis and preparation of the manuscript. JIP was involved in statistical analysis and preparation of the manuscript. EA developed and conducted the search strategy. MC contributed to data extraction, AU to statistical analysis and AQ to analysis and preparing of the manuscript. All authors critically revised the manuscript and approved it for publication. AA-R is the guarantor.

## Pre-publication history

The pre-publication history for this paper can be accessed here:


